# Leistenhernienoperationen – immer ambulant?

**DOI:** 10.1007/s00104-023-01818-9

**Published:** 2023-02-14

**Authors:** DACH-Konsensusgruppe ambulante Leistenhernienchirurgie, H. Niebuhr, F. Köckerling, R. Fortelny, H. Hoffmann, J. Conze, R. G. Holzheimer, A. Koch, G. Köhler, C. Krones, J. Kukleta, A. Kuthe, B. Lammers, R. Lorenz, F. Mayer, M. Pöllath, W. Reinpold, R. Schwab, B. Stechemesser, D. Weyhe, M. Wiese, K. Zarras, H.-J. Meyer

**Affiliations:** Hamburger Hernien Centrum, Eppendorfer Baum 8, 20249 Hamburg, Deutschland

**Keywords:** DACH-Region, Ambulantes Operieren, Vergütung, Patientensicherheit, Risikofaktoren, DACH region, Outpatient surgery, Remuneration, Patient safety, Risk factors

## Abstract

Leistenhernienoperationen stellen mit ca. 300.000 Eingriffen jährlich in Deutschland, Österreich und der Schweiz (DACH-Region) die häufigste Operation überhaupt dar. Trotz des bekundeten politischen Willens und des zunehmenden Drucks der Gesetzgeber zur Vermeidung kostenintensiver stationärer Behandlungen, so viele Operationen wie möglich ambulant durchzuführen, spielt in der DACH-Region die ambulante Versorgung bislang nur eine untergeordnete Rolle. Die Vorstände der Fachgesellschaften Deutsche Hernien Gesellschaft (DHG), Chirurgische Arbeitsgemeinschaft Hernien (CAH der DHG), Österreichische Hernien Gesellschaft (ÖHG) und Schweizerische Arbeitsgruppe für Hernienchirurgie (SAHC) setzen sich mit diesem Problem auseinander, schildern die Ausgangslage und bewerten die aktuelle Situation.

## Abstract (en)

Inguinal hernia operations represent the most frequent operations overall with 300,000 interventions annually in Germany, Austria and Switzerland (DACH region). Despite the announced political willingness and the increasing pressure from the legislator to avoid costly inpatient treatment by carrying out as many outpatient operations as possible, outpatient treatment has so far played a subordinate role in the DACH region. The Boards of the specialist societies the German Hernia Society (DHG), the Surgical Working Group Hernia (CAH of the DHG), the Austrian Hernia Society (ÖHG) and the Swiss Working Group Hernia Surgery (SAHC) make inroads into this problem, describe the initial position and assess the current situation

## Hintergrund

Seit vielen Jahren besteht in Deutschland, Österreich und der Schweiz (DACH-Region) der politische Wille, so viele Operationen wie möglich ambulant durchzuführen [5, 9, 25, 52]. Vor dem Hintergrund der dominierenden Ökonomisierung im Gesundheitssystem, erhofft man sich dadurch signifikante Einsparungseffekte, die durch die Vermeidung der kostenintensiven stationären Behandlung erzielt werden sollen. Auch die Operation der Leistenhernie, die mit ca. 300.000 Eingriffen jährlich in der DACH-Region die häufigste Operation überhaupt darstellt, soll möglichst ambulant erfolgen.

In der DACH-Region ist der Anteil ambulant operierter Leistenhernien jedoch seit vielen Jahren unverändert gering, es zeichnet sich sogar ein abnehmender Trend ab. In einer kürzlich publizierten Analyse von 340.000 Leistenhernienoperationen aus dem Herniamed-Register, nahm der Anteil ambulant operierter Leistenhernien im Zeitraum von 2013 bis 2019 von 20 % auf 14 % ab [24]. In Österreich beträgt der Anteil ambulant durchgeführter Leistenhernieneingriffe ca. 5 %, in der Schweiz geschätzt weniger als 15 %.

Damit steht die Entwicklung in der DACH-Region den Trends anderer Länder und den politischen Forderungen diametral entgegen, weshalb sich die Vorstände der Fachgesellschaften Deutsche Hernien Gesellschaft (DHG), Chirurgische Arbeitsgemeinschaft Hernien (CAH der DHG), Österreichische Hernien Gesellschaft (ÖHG) und Schweizerische Arbeitsgruppe für Hernienchirurgie (SAHC) veranlasst sehen, sich mit dem Problem auseinanderzusetzen.

## Ausgangslage in der DACH-Region

*In Deutschland* werden pro Jahr ca. 250.000 Leistenhernien operiert. Ob eine Leistenhernie ambulant versorgt werden kann oder nicht, wird durch die Kriterien des G‑AEP (Gesundheit Appropriateness Evaluation Protocol) und den AOP-Katalog (Katalog ambulant durchführbarer Operationen) festgelegt [3]. Darin sind z. B. Schwere der Erkrankung, Aspekte der Operation und Komorbiditäten definiert, die eine ambulante Operation erlauben bzw. ausschließen. Die organisatorischen Voraussetzungen für ambulantes Operieren inkl. der Qualitätssicherungsmaßnahmen sind im Sozialgesetzbuch (SGB) geregelt. Zudem gibt es Bestrebungen zur Erweiterung des ambulanten Operierens unter Hinzunahme einer vergütungsrelevanten Schweregraddifferenzierung [1].

Die Vergütung der ambulanten Hernienoperation ist durch den Einheitlichen Bewertungsmaßstab (EBM) für ärztliche Leistungen geregelt. Dieser ist jedoch bei der Leistenhernie hochgradig defizitär. So beträgt z. B. die Vergütung für die Versorgung einer einseitigen Leistenhernie im ambulanten Bereich nur 25 % der Vergütung einer stationären Versorgung.

In einem aktuellen Ergebnisbericht (11/2022) des Deutschen Krankenhausinstituts zum Thema „Ambulantes Operieren im Krankenhaus“ kommen die Expertinnen und Experten zu dem Schluss, dass die auf dem EBM basierenden Vergütungsstruktur kritisch hinterfragt werden muss, die strukturellen Unterschiede zwischen Krankenhäusern und Vertragsärzten in der Vergütung nicht berücksichtigt sind und ambulantes Operieren allgemein deutlich unterfinanziert ist [66].

*In Österreich* werden in der DACH-Region die wenigstens Leistenhernien ambulant versorgt. Von ca. 22.000 Leistenhernienoperationen pro Jahr betrug der Anteil der ambulant durchgeführten Leistenhernienoperationen in den letzten drei Jahren lediglich 5–7 %.

Zwei Aspekte können das erklären. Zum einen wurde die Vergütung der ambulant versorgten Leistenhernien im Jahr 2020 signifikant herabgesetzt. Im Vergleich zur Vergütung in stationärer Versorgung schlagen für die ambulante TAPP/TEP (transabdominal-präperitoneal/total extraperitoneal) ein Abschlag von 880 € bei Patientinnen und Patienten über 64 Jahre zu Buche. Diese finanzielle Mindervergütung von bis zu 54 % der stationären Vergütung für die Erbringung einer identischen Operationsleistung konterkariert das Bestreben, Leistenhernienoperationen verstärkt ambulant zu erbringen. Zum anderen ist die notwendige Infrastruktur (z. B. Tagesklinik) in den meisten Krankenhäusern Österreichs nicht vorhanden. Vor dem Hintergrund des finanziellen Defizits im ambulanten Setting fehlt hier auch der betriebswirtschaftliche Anreiz für die Krankenhäuser, in den kostenintensiven Aufbau einer Tagesklinik zu investieren und somit die stationäre Versorgung zugunsten der ambulanten Versorgung zu verlassen.

*In der Schweiz* werden pro Jahr ca. 20.000 Leistenhernien operiert, den Anteil ambulanter Operationen schätzt die SAHC auf weniger als 15 %. In der seit 2019 bestehenden AVOS-Liste (Ambulant-Vor-Stationär) schreibt der Gesetzgeber vor, wann die Leistenhernienoperation ambulant erfolgen muss. Es sind zwar Ausnahmekriterien definiert, die jedoch viele bekannte Risikofaktoren nicht berücksichtigen. Organisatorische oder bauliche Voraussetzungen für ambulantes Operieren sind nicht genau festgelegt.

Die Vergütung der ambulanten Operation erfolgt durch eine Tarifstruktur aus den 1990er-Jahren (TARMED), in der die Leistenhernienoperation hochgradig defizitär bleibt. So ergibt die ambulante einseitige Leistenhernienoperation nur 30–35 % des Ertrags einer stationär durchgeführten Operation. Zusätzlich ergibt sich in der Schweiz die Situation, dass die ambulante Operation einer Leistenhernie für die Krankenkassen 10–15 % teurer ist als die stationäre Operation, da der Kanton die stationäre Behandlung zu mehr als 50 % mitfinanziert, die ambulante Versorgung jedoch nicht [47].

## Bewertung der aktuellen Situation

### Fehlende Berücksichtigung der bekannten Risikofaktoren

Ein kürzlich in Deutschland publiziertes Gutachten mit dem Auftrag, eine „möglichst umfassende Ambulantisierung“ zu begründen (IGES-Gutachten) hat für Aufsehen gesorgt, denn es enthält aus hernienchirurgischer Sicht mehrere diskussionswürdige Aspekte [1, 3].

Das Gutachten hält z. B. als höchstes Ziel fest, dass die Patientensicherheit rund um die Operation gegeben sein muss. Zitiert aus dem Gutachen:

„Würde hingegen eine substanzielle Erweiterung des AOP-Kataloges auf einer stärker generalisierenden Einstufung von Leistungen basieren, bestünde ein höheres Risiko der Gefährdung von Patientensicherheit.“

Das Gutachten betont daher, dass die Zuordnung der geplanten Operation zu den drei möglichen Versorgungsformen (ambulant – kurzstationär – stationär) eine rein ärztliche Entscheidung ist und bleiben muss, die die operationsspezifischen Risikofaktoren, aber auch die sozialen und gesundheitlichen Begleitumstände der Patientinnen und Patienten berücksichtigt [1]. Diese Prämisse wird in der jetzigen Konstellation des ambulanten Operierens nicht nur in Deutschland, sondern auch in der gesamten DACH-Region nicht ausreichend berücksichtigt.

Die Patientensicherheit ist unbestritten dann gefährdet, wenn z. B. bestimmte Risikofaktoren für Komplikationen in der Hernienchirurgie bestehen, die jedoch in dem Entscheidungsprozess für oder gegen eine ambulante Operation nicht berücksichtigt werden dürfen.

Auffällig ist, dass in den Vorgaben zum ambulanten Operieren in Deutschland (G-AEP und AOP-Katalog) und der Schweiz (AVOS-Liste) viele Risikofaktoren nicht berücksichtigt sind und somit ein Wechsel in die stationäre Behandlung bei bestimmten Risikokonstellationen nicht möglich ist.

Als Beispiel sei hier die fehlende obere Altersbegrenzung oder eine bestehende Medikation mit bestimmten Thrombozytenaggregationshemmern in der AVOS-Liste in der Schweiz genannt. Sofern nicht gravierende Systemerkrankungen vorliegen, müssen z. B. auch betagte über 90-jährige Patientinnen und Patienten ambulant versorgt werden, obwohl Patientinnen und Patienten in dieser Altersgruppe – unabhängig von Systemerkrankungen – ein signifikant höheres Komplikationsrisiko haben. Gleiches gilt für das bekannte höhere Nachblutungsrisiko bei Thrombozytenaggregationshemmern.

In den Tab. 1, 2 und 3 sind die operationsspezifischen und patientenbezogenen Risikofaktoren für Leistenhernienoperationen und deren Einfluss auf den peri- und postoperativen Verlauf aufgelistet. Diese gelten sowohl für in Praxen als auch in Krankenhäusern durchgeführte ambulante Operationen gleichermaßen. Berücksichtigt werden muss auch der unmittelbare peri- und postoperative Verlauf inkl. des subjektiven Befindens der Patientinnen und Patienten nach der Operation mit der Option des Abweichens vom vorgesehenen ambulanten Setting. Die Anwendung der in Tab. 1 und 2 definierten Kriterien ermöglicht damit eine von finanziellen und politischen Aspekten unabhängige, sichere Zuordnung zu den drei Versorgungsstufen, die allein auf chirurgisch-wissenschaftlicher Evidenz basieren und somit die Patientensicherheit aufrechterhalten.

**Table 1:** 

Risikofaktor	Beeinflussung Outcome
*Hohes Alter*	*Alter >* *80 Jahre*
	Mehr Komplikationen und Reoperationen [37]
	Bei bestehenden Komorbiditäten vervielfacht sich das Letalitätsrisiko [43]
	Mehr Serome [44], Harnverhalte und Readmission [62]
	*Alter >* *60 Jahre*
	Mehr Harnverhalte
	Mehr Komplikationen [31, 64]
*ASA III und höher*	Mehr Komplikationen und Reoperationen [37, 64]
	Höheres Risiko für Letalität [43]
*Geschlecht*	Frauen mit höherem Risiko für Schmerzen [21, 64]
*Adipositas*	Tendenz zu mehr Komplikationen bei Adipositas [7]
*COPD*	Signifikant mehr Komplikationen [23]
	Erhöhte Letalität in der tagesklinischen Chirurgie [36]
	Risikofaktor bezüglich ungeplanter Verlängerung der postoperativen Aufenthaltsdauer [60]
*Kortison*	Die kombinierte postoperative Medikation mit Kortison (Betamethason) und NSAR (Diclofenac) erhöht das postoperative Blutungsrisiko in der Hernienchirurgie [50, 51, 59]
*Diabetes mellitus*	Unabhängiger Risikofaktor für postoperative Komplikationen [12, 23]
*Gerinnungsstörung* *Antikoagulation* *Thrombozytenaggregationshemmer* *NOAK* *OAK*	Patientinnen und Patienten mit antithrombotischer oder gerinnungshemmender Medikation haben ein 4‑fach erhöhtes postoperatives Blutungsrisiko [19]
	Auch nach Absetzen von TAH, NOAK und Marcumar mit Bridging besteht ein deutlich erhöhtes Risiko von Blutungskomplikationen
	Gerinnungsstörung erhöht postoperative Komplikationen, Reoperationsrate und Gesamtkomplikationsrate [23, 29, 39, 45, 49]
	Deutliche Zunahme der Blutungskomplikationen [7, 12, 21, 23, 36, 59, 60, 64]
*Immunsuppression*	Immunsuppression erhöht das Risiko für ein Rezidiv [27] – keinen Einfluss auf postoperative Wundmorbidität [61]
*Leberzirrhose*	Deutliche Zunahme der Komplikationsraten [15, 37, 43, 62]
*Nikotinabusus*	Deutliche Zunahme der allgemeinen und chirurgischen Komplikationsraten [31, 62]

*ASA* American Society of Anaesthesiologists,* COPD* „chronic obstructive pulmonary disease“, *NOAK* neue orale Antikoagulanzien,* NSAR* nichtsteroidale Antirheumatika, *TAH* Thrombozytenaggregationshemmer

**Table 2:** 

Risikofaktor	
*Beidseitige Hernie*	*Erhöhtes perioperatives Risiko* daher keine prophylaktische Operation einer gesunden Leiste (aber eher stationäres Vorgehen; [10, 17, 33, 41])
*Inkarzerierte Hernie*	*Inkarzerierte Hernien* gelten als Notfälle und sind daher* stationär* zu behandeln. Aufenthaltsdauer: 2,0–5,4 Tage. Komplikationsraten: 20,2–40,0 % [8, 16, 28, 30, 34]
*Rezidivhernie*	Ein signifikant ungünstiger Zusammenhang mit der gesamten perioperativen Komplikationsrate zeigte sich für *Rezidiveingriffe,* beidseitige Versorgung, hohes Alter, ASA-Score III/IV, Schenkelhernie, antithrombotische Medikation, Rauchen, COPD und Kortikosteroidmedikation [23, 54]
*Rezidivhernie 2 Herniasurge Guidelines*	Herniasurge: Da das Komplikationspotenzial der offenen Rezidivleistenhernienreparation – einschließlich Hodenatrophie und/oder Nerveneinklemmung und -schädigung – höher ist als bei der primären Rekonstruktion, empfehlen wir dringend, dass diese Operation von einem erfahrenen Hernienchirurgen durchgeführt wird
	Schlussfolgerung: Angesichts der oben genannten Faktoren bleibt die Behandlung rezidivierender und wiederholt rezidivierender Leistenhernien eine Herausforderung. Es kann möglich sein, die Anzahl von Rezidiven und andere Komplikationen zu minimieren, indem eine geeignete Operationstechnik verwendet wird, die vorherigen Ansätze berücksichtigt werden und erfahrene Hernienchirurgen hinzugezogen werden, um diese Fälle zu behandeln [6, 11, 23, 26, 32, 35, 54]
*Schmerz präoperativ*	Präoperativer Schmerz führt zu akuten (aPIP) und chronischen Leistenschmerzen (cPIP; *Komplikation)* *Beim Auftreten der Komplikation ggf. stationäre Behandlung erforderlich* [22, 38, 40, 42, 46, 55]
*Schmerz postoperativ*	Starker akuter postoperativer Schmerz führt zu chronischen postoperativen Leistenschmerzen (cPIP; *Komplikation) und muss ggf. mit einer Revisionsoperation behandelt werden (stationäre Behandlung)*. (Siehe auch Tab. 3: Postoperativer Schmerz; [40, 46, 55])
*Technisches Risiko TAPP/TEP*	TAPP und TEP zeigen ein erhöhtes intraoperatives Risiko bei insgesamt niedriger Inzidenz,* aber beim Auftreten z.* *B. einer retroperitonealen Blutung lebensgefährlich und daher nicht ambulant durchzuführen*. (Siehe auch Tab. 3: Gefäßverletzungen; [14, 48, 63])
*Antikoagulation und Operationstechnik*	Patientinnen und Patienten unter Therapie mit Thrombozytenaggregationshemmern oder Antikoagulanzien werden signifikant häufiger offen-chirurgisch als laparoendoskopisch versorgt; (OR = 1,2; 95 %-CI 1,1–1,4 und OR = 1,4; 95 %-CI 1,3–1,5; [2])

*aPIP CI* Konfidenzintervall, *ASA* American Society of Anaesthesiologists,* cPIP* „chronic postoperative inguinal pain“, *OR* Odds Ratio, *TAPP* transabdominelle präperitoneale Patch-Technik, *TEP* Totalendoprothese

**Table 3:** 

**Intraoperative Faktoren**	
*Gefäßverletzungen*	Kommen nach Angaben der Herniasurge-Gruppe [56] in 1,39 % der Fälle nach einer TEP und in 1,13 % der Fälle nach bzw. bei einer TAPP vor. Die Daten beziehen sich auf eine Herniamed-Auswertung [18]
*Verletzungen des Darmes* bzw. *der Harnblase*	Kamen in 0,27 % bei der TAPP und in 0,1 % der Fälle bei der TEP vor. Bittner et al. [4] konnten für die TAPP eine Verletzung viszeraler Organe von 0,21 % vs. TEP 0,11 % aufzeigen und eine Gefäßverletzungsrate von 0,25–0,42 %
**Faktoren in der frühen postoperativen Phase (AWR)**	
*Nachblutung*	Eine Nachblutung ist oft erst Stunden, gelegentlich auch Tage nach der Indexoperation klinisch bemerkbar (Hb-Abfall, Blutdrucksenkungen, Drainagemengen, sichtbare Hämatome). Eine 100 %ige Sicherheit kann es diesbezüglich daher niemals geben. Sollten allerdings bereits in der frühen postoperativen Phase Indikatoren wie der Blutdruck auf eine Komplikation hinweisen, verbietet sich eine ambulante Weiterbehandlung und eine umgehende bildgebende Diagnostik muss veranlasst werden. Nachblutungen treten in der Herniamed-Auswertung in 1,12 % der Fälle auf, bei Patientinnen und Patienten mit Antikoagulation in 3,91 % [19]
*Postoperativer Schmerz*	Postoperativer Schmerz ist ein Indikator für die Entwicklung chronisch postoperativer Schmerzen [3], bei akuten Schmerzen, die nicht oder nur schwer mit Analgetika unter VAS 4 zu kupieren sind, sollte daher von einer ambulanten Weiterbehandlung abgesehen werden. Bei starken postoperativen Schmerzen sollte ggf. eine sofortige Revision erwogen werden, die ein ambulantes Vorgehen eher schwierig macht [20]
*Postoperative Bauchschmerzen*	Und/oder Wundschmerzen, die über das Maß leichter bis moderater Wundschmerzen hinausgehen, können erstes Symptom einer ernsten Komplikation wie Nachblutung, Darmverletzung, Darmverschlusses, foudroyante Infektion sein und sind daher zwingend engmaschig klinisch zu kontrollieren, was nur stationär möglich ist [4, 13, 18–20, 40, 56]

*AWR* Aufwachraum, *TAPP* transabdominelle präperitoneale Patch-Technik, *TEP* Totalendoprothese, *VAS* visuelle Analogskala

### Gefährdung der chirurgischen Weiterbildung

Die Leistenhernienoperation ist ein klassischer Ausbildungseingriff. Die finanziellen Defizite, wie sie bei der ambulanten Leistenhernieoperationen in Deutschland, Österreich und der Schweiz generiert werden, gefährden systematisch die chirurgische Ausbildung. Eine Ausbildungsoperation dauert aufgrund der noch geringen operativen Erfahrung der jungen Kolleginnen und Kollegen deutlich länger. Zudem sollte stets genug Zeit für Instruktionen, Besprechung von Zwischenschritten und ein Debriefing vorgesehen sein. Aufgrund des aktuell unvermeidlichen Defizits bei ambulanter Chirurgie sind nun immer mehr Krankenhäuser dazu übergegangen, die ambulante Leistenhernienoperationen zu einem großen Teil nur noch durch erfahrene Operateure (in Deutschland geforderter Facharztstatus bei ambulanten Operationen) und oft ohne Assistenten durchführen zu lassen. Damit soll durch kürzere Operationszeiten und schnelleres „Durchschleusen“ der Patientinnen und Patienten das finanzielle Defizit etwas abgemildert werden. Die Ausbildung des chirurgischen Nachwuchses wird dadurch erheblich erschwert.

### Was können wir aus anderen Ländern lernen?

Im Gegensatz zur DACH-Region ist die ambulante Leistenhernienchirurgie in vielen anderen Ländern der westlichen Welt etablierter Standard. Die Gründe für diese Entwicklung sind vielfältig. In den Niederlanden und einigen skandinavischen Ländern war vor allem eine knappe Anzahl stationärer Betten der Antrieb dafür, mehr Operationen in den ambulanten Sektor zu verlagern [25]. In anderen Ländern waren die zu erzielenden globalen Kosteneinsparungen die Triebfeder für diese Entwicklung [5].

Eine von uns durchgeführte Umfrage bei den nationalen Herniengesellschaften ergab, dass in den USA, Großbritannien, Italien, Finnland, Dänemark, Schweden, Belgien und Spanien der Anteil ambulanter Leistenhernienoperationen weit über 90 % und in Schweden bei 81 % liegt [5, 52, 53]. Die Umfrage ergab auch, dass ein Abweichen vom ambulanten Operieren eine rein ärztliche Entscheidung ist, welche u. a. auf medizinischen oder sozialen Risikofaktoren beruht. Verbindliche gesetzliche Vorschriften, wie sie in Deutschland (G-AEP) und der Schweiz (AVOS-Liste) etabliert wurden, existieren in diesen Ländern nicht.

Das Erreichen einer hohen Quote ambulanter Operationen entsteht nicht aus sich selbst heraus. Die hohe Akzeptanz in den „Erfolgsländern“ erklärt sich nicht allein mit kulturellen Unterschieden zur DACH-Region, vielmehr wurden dort gezielt Anreize gesetzt. Beispielsweise wird ambulantes Operieren in fast allen „Erfolgsländern“ mindestens genauso gut vergütet wie eine stationäre Operation. Der globale Einsparungseffekt wird dann durch die Schonung der teuren stationären Infrastruktur erreicht und nicht mit der Unterfinanzierung der eigentlichen Leistungserbringung. Im NHS (National Health Service), wo aktuell 10 % der Bevölkerung auf eine Operation warten, wurde das ambulante Operieren einige Zeit sogar besser vergütet als die stationäre Operation, um die Entwicklung hin zum ambulanten Operieren anzustoßen [57]. Ein weiterer Erfolgsfaktor ist das Vorhalten einer Infrastruktur, die es jederzeit ermöglicht, vom ambulanten in den stationären Sektor zu wechseln [65].

Die Erfolgsgeschichte des ambulanten Operierens war daher vor allem möglich, weil bestimmte Anreize gesetzt und Voraussetzungen in Infrastruktur und Management erfüllt wurden [5, 58]. Dazu gehören u. a. der Aufbau entsprechender Infrastruktur (Tageskliniken), die räumliche und operative Trennung von ambulanten und stationären Behandlungspfaden und der Abbau regulatorischer und ökonomischer Barrieren. Gerade im Bereich der in Deutschland im Krankenhaus durchgeführten ambulanten Operationen sind hierzu noch viele Fragen ungelöst.

## Schlussfolgerung

Zusammenfassend spielt die ambulante Leistenhernienchirurgie trotz des bekundeten politischen Willens und zunehmenden Drucks der Gesetzgeber in der DACH-Region bislang nur eine untergeordnete Rolle.

Als wichtigster Kritikpunkt muss die durch den Gesetzgeber zunehmende Einschränkung der ärztlichen Hoheit bei der Entscheidung für oder gegen eine ambulante Operation genannt werden. Insbesondere ist durch die fehlende Möglichkeit der Berücksichtigung wichtiger Risikofaktoren (z. B. hohes Alter, Adipositas) aus unserer Sicht die Patientensicherheit gefährdet, für die Chirurginnen und Chirurgen schlussendlich verantwortlich sind (Tab. 1, 2 und 3).

Der Zusammenhang zwischen Komorbidität als Risikofaktor und dem Auftreten von Komplikationen nach Leistenhernienoperationen ist in allen Hernienregisterstudien sowie in der Literaturdurchsicht als stark evident einzustufen. Einige Risikofaktoren finden in den Ausnahmekriterien vom ambulanten Operieren keine Berücksichtigung, ein Umstand, der die Patientensicherheit gefährden kann. Daher müssen die definierten präoperativen Risikofaktoren, der intraoperative Verlauf und soziale Faktoren als objektive Kriterien zur Planung einer ambulanten oder stationären Operation herangezogen werden (Tab. 1 und 2).

Wesentliche Aspekte sind neben den oben beschriebenen präoperativen Risikofaktoren auch die im Verlauf einer Behandlung möglicherweise hinzutretenden Faktoren:

- intra- und
- postoperative Komplikationen,
- Reoperation,
- allgemeine Komplikation,

die als Grundlage dienen, die ex ante getroffene Zuordnung zu einer der drei Versorgungsstufen im Versorgungsverlauf im Sinne eines Strategiewechsels abändern zu können (Tab. 3).

Ein weiterer Aspekt ist der fehlende Anreiz, den Anteil ambulanter Leistenhernienoperationen zu erhöhen. Ganz im Gegenteil, in Deutschland, Österreich und der Schweiz mangelt es an einer kostendeckenden Vergütung im ambulanten Bereich. In einer Vielzahl von Einrichtungen mangelt es an der notwendigen Infrastruktur.

## Folgerungen

Oberstes Ziel einer jeden Operation ist die Aufrechterhaltung der Patientensicherheit. Die Zuteilung von Patientinnen und Patienten zu den einzelnen Versorgungsstufen (ambulant/kurz-stationär/stationär) muss daher unabhängig von Vorgaben der Gesetzgeber und wirtschaftlichen Aspekten allein auf der Grundlage der Berücksichtigung der Risikofaktoren, intraoperativen Faktoren und sozialen Faktoren wieder die Entscheidung der Chirurgin bzw. des Chirurgen werden.

Es muss jederzeit die Möglichkeit des Wechsels der gewählten Versorgungsstufe unter Beachtung des peri- und unmittelbar postoperativen Verlaufs bestehen.

Um eine größere Anzahl von Patientinnen und Patienten ambulant versorgen zu können, ist die flächendeckende Bereitstellung baulicher, personeller und organisatorischer Infrastruktur erforderlich.

Ambulantes Operieren kann aktuell in Deutschland, Österreich und der Schweiz nicht kostendeckend durchgeführt werden. Eine Gleichstellung der Leistungsvergütung ambulanter und stationärer Operationen ist daher erforderlich.

Die Unterfinanzierung des ambulanten Operierens gefährdet die Qualität der chirurgischen Ausbildung. Auch deshalb ist eine gleichwertige Vergütung von ambulanter und stationärer Operation erforderlich.
